# Zebrafish aversion to infrasound in an open field test

**DOI:** 10.3389/fnbeh.2022.1019368

**Published:** 2023-01-06

**Authors:** Kale R. Scatterty, Taylor Pitman, Tristan Eckersley, Rodney Schmaltz, Trevor J. Hamilton

**Affiliations:** Department of Psychology, MacEwan University, Edmonton, AB, Canada

**Keywords:** behaviour, locomotion, acoustics, *Danio rerio*, aversion

## Abstract

Aquatic species are capable of detecting infrasound (sub-20 Hz frequencies) which may be a source of anthropogenic pollution and have a detrimental impact on the environmental fitness of fish. Infrasound is generated by infrastructure, producing acoustic frequency peaks that are not discernible by humans. The presence of these frequencies may therefore impact the environmental wellbeing of aquatic laboratory animals, which are often housed in spaces adjacent to facilities producing infrasound. To investigate the potential impact of infrasound, we used wild-type zebrafish (*Danio rerio*) and exposed them to short periods of infrasound at either 5, 10, 15, or 20 Hz, or 0 Hz as a control group. A motion-tracking software system was used to monitor fish movement in an open field test and arena location, distance moved, and immobility were quantified. There was a significant effect of 15 Hz which caused the fish to spend more time away from the infrasound source. The 20 Hz group also spent significantly less time in the zone closest to the speaker. There were no differences in distance moved or immobility between infrasound and control groups. These findings demonstrate that 15 Hz infrasound has aversive effects on zebrafish, causing them to move away from the sound source. To enhance environmental enrichment and wellbeing of aquatic laboratory animals, sources of infrasound pollution should be investigated and mitigated.

## Introduction

Human created infrastructure and technology are rapidly developing, as are concerns of their impact on local environments and ecosystems. Infrasound pollution has become a growing area of interest as it may affect the welfare of humans and laboratory organisms within urban structures. Infrasound frequencies are acoustic signals that represent soundwaves oscillating below the human hearing threshold (i.e., sub-20 Hz) (Boczar et al., 2022). Mechanical ventilation and air conditioning systems can cause elevated levels of infrasound (Burt, 1996; Salt and Hullar, 2010), as can energy, heating, and other mechanical systems commonly found throughout many buildings (Salt and Hullar, 2010; Persinger, 2013). Local urban transportation, such as automobile and railroad/light rail transit, can also increase levels of low frequency noise, including infrasound frequencies, most significantly at the street level of urban buildings (Butkus and Vasiliauskas, 2013). Since many animal research laboratories are commonly found in the ground or basement floors, these factors warrant evaluation of the effects of these frequencies on laboratory animals.

Many teleost fish species are sensitive to infrasound frequencies, such as Atlantic Cod (*Gadus morhua*), Perch (*Sebastes norvegicus*), and Salmon (*Salmo salar*) *via* the otolithic organs of their inner ear (Karlsen, 1992b; Enger et al., 1993; Sand and Karlsen, 2000). Infrasound perception in these fish has been hypothesized to contribute to spatial navigation and predatory and prey responses, particularly in mediating escape behaviours (Sand and Karlsen, 1986, 2000). In fish sensitive to infrasound, some species are noted to exhibit aversive and anxious behaviours in response to infrasound frequencies. Atlantic Cod increase avoidance behaviour in the presence of 12.5 Hz frequencies (Bui et al., 2013); startle responses to infrasound have been observed in *Rutilus rutilus* (Karlsen et al., 2004); avoidance responses can be conditioned into Perch using infrasound stimuli (Karlsen, 1992b; Sand and Karlsen, 2000); and fear responses can be conditioned into Plaice (*Pleuronectes platessa*) with 0.1–30 Hz tones (Karlsen, 1992a). Spatial avoidance responses appear to be the strongest among these species in the presence of infrasound between 5 and 15 Hz (Sand et al., 2001). These studies generated their infrasound stimuli within the testing water column, however, it is unknown how infrasound generated above the water can influence fish behaviour.

Zebrafish (*Danio rerio*), a small freshwater teleost species, is a growing model organism for studying anxiety-like and avoidance behaviours using environmental and pharmacological manipulation. With pronounced behavioural phenotypes for fear and aversion responses, ease of use and cost effectiveness in behavioural research, and comparable performance in well-established mammalian paradigms of anxiety testing (Maximino, 2011; Kalueff et al., 2013), zebrafish are an ideal model organism for studying potential anxiogenic and aversive effects of infrasound. Previous pharmacological research has established zebrafish as an effective model organism of anxiety, using various compounds like scopolamine and ethanol (Holcombe et al., 2013; Hamilton et al., 2017). Given that zebrafish share many physiological and anatomical similarities with other teleost species in auditory perception (Enger et al., 1993; Sand and Karlsen, 2000; Yao et al., 2016), it follows that a zebrafish model of infrasound-induced behaviours could provide a useful tool in behavioural neuroscience and other biological sciences as a detection and evaluation method in habitat enrichment and animal wellbeing efforts.

In this study, we exposed naïve adult wild-type zebrafish to tonal infrasound frequencies (5, 10, 15, and 20 Hz) generated outside of the water column to evaluate their impact on behaviour. An open field test was used to assess the potential anxiogenic or aversive effects of infrasound on zebrafish locomotion and location preference. The open field test has been shown previously to be an effective evaluation method in behavioural research for the measurement of anxiety-like and aversive behaviour in zebrafish across various well-established paradigms (Blaser et al., 2010; Maximino et al., 2010; Stewart et al., 2012; Szaszkiewicz et al., 2021). This test allows for quantification of anxiety-like and aversive behaviour with motion tracking software using artificial zones acting as proxies for anxiety and aversion. In this study, three virtual zones perpendicular to the direction of incoming infrasound were used to quantify the potential aversion. A thigmotaxis zone was created virtually near the arena wall to quantify “wall-hugging” behaviour, an indicator of anxiety. We hypothesized that naïve wild-type zebrafish would exhibit behaviours indicative of aversive and anxiety-like behaviour in response to infrasound frequencies between 5 and 15 Hz.

## Materials and methods

### Animals and housing

Adult wild-type zebrafish (*Danio rerio*, *n* = 155, ∼50:50 male-female, 8–12 months old) were obtained commercially from Aquatic Imports (Calgary, AB, Canada). The zebrafish were housed in an Aquatic Habitats (AHAB, Aquatic Ecosystems, Inc. Apopka, FL, USA) three-shelf bench top system. Daily husbandry procedures were carried out as described previously (Holcombe et al., 2013), including maintenance of pH at 6.5–8.0 and temperature at 26–28°C. The habitat photoperiod rotated on an alternating 12-h light and dark cycle and fish were fed New Life Spectrum Small Fish Formula dry fish pellets (New Life International Inc., Homestead, FL, USA) once per day. Feeding on experimental days took place after testing (Hamilton et al., 2017). On experimental days, each zebrafish was tested only once and was naïve to behavioural testing. All experiments were approved by MacEwan University’s Animal Research Ethics Board (AREB) under protocol number 05-12-13 in compliance with the Canadian Council for Animal Care (CCAC) guidelines and regulations for care and use of experimental animals. All experiments also adhered to the ARRIVE guidelines for animal research. The authors of this study affirm that all experiments and procedures involved conformed to CCAC guidelines and regulations.

### Infrasound administration

**FIGURE 1:**
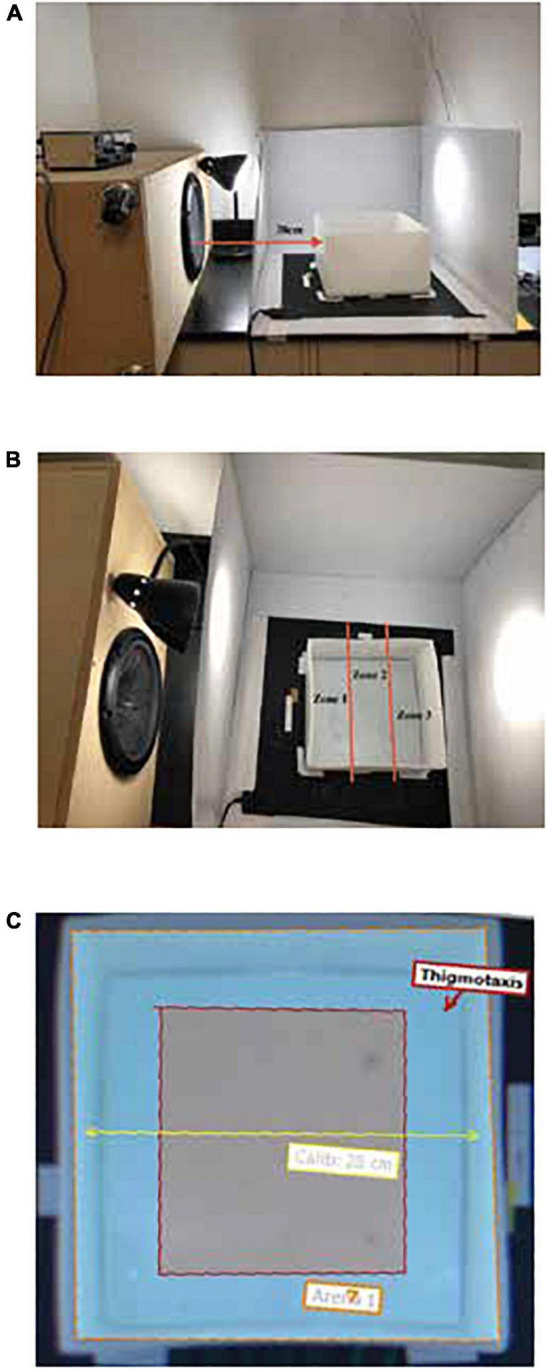
Infrasound speaker placement and EthoVision XT digital zone layouts in the open field test. **(A)** Infrasound speaker placement of 38 cm to the left of the testing arena. **(B)** Location preference zones by distance from the infrasound source (Zone 1 = Speaker; Zone 2 = Middle; Zone 3 = Far). **(C)** Thigmotaxis zone measured 4.8 cm from the water line of each arena wall.

Infrasound was generated *via* a 12″ Pyle subwoofer speaker oriented towards the testing arena ∼38 cm away (Figure 1A). Zebrafish were exposed to infrasound frequencies at levels of 0, 5, 10, 15, and 20 Hz with a constant standardized amplitude of ∼60 dB. These amplitudes are just below those created by mechanical energy, ventilation, and heating sources, which are often ∼70–80 dB at a distance of 100 meters but can be 10–30 dB higher if the source is downwind or within a sealed environment (Jakobsen, 2005; Salt and Hullar, 2010; Butkus and Vasiliauskas, 2013; Persinger, 2013). As a precaution for the research team, an amplitude of up to 65 dB was used which is considered safe for exposure in humans over prolonged periods,^1^ but hearing protection was still implemented. Infrasound stimuli were recorded in various locations within and around the testing arena using a Spider-20 microphone with Spider EDM 6.0 software (Crystal Instruments, Santa Clara, CA, USA) to validate frequencies and amplitude. Control data was collected on each infrasound testing day to ensure that control fish were tested under identical conditions to all experimental fish. All factors, such as lighting (∼32 cad/m^3^) and arena water temperature (25–28°C), remained constant across control and experimental conditions. Researchers were not blinded to treatment groups, but all experiments were conducted under identical conditions and analysed with motion tracking software.

### Procedure

#### Open field test

The open field test is a frequently used method of assessing zebrafish behaviour and locomotion and can be used to measure aversion and anxiety-like responses *via* changes in location preference. This was chosen as an adaptation of the designs of Sand and Karlsen (1986, 2000), Karlsen (1992a,b), and Karlsen et al. (2004) which also utilized top-down measurement of horizontal fish movement. Zebrafish behaviour and locomotion were assessed across virtual zones in a testing arena with the infrasound speaker oriented as seen in Figure 1B. The open field test arena was a 28 cm × 28 cm plastic tank with a height of 15 cm. The arena was divided into three virtual zones—Zone 1 (Speaker), Zone 2 (Middle), and Zone 3 (Far)—as well as a 4.8 cm thigmotaxis zone from the inside perimeter as seen in Figure 1C. The tank was filled to a depth of 6 cm with habitat water which was changed every 3 trials. Water temperature was maintained between 25 and 28°C for all trials.

#### Infrasound exposure

Zebrafish were placed in the center of the arena and allowed to acclimate for a trial period of 5 min. During this time fish movement throughout the arena was recorded to test whether there were any baseline differences in fish across groups. Immediately after the acclimation trial period, experimental manipulation began, specific to each condition group. The groups were exposed to 5 min of either 0 (control), 5, 10, 15, or 20 Hz frequencies. All groups consisted of *n* = 31 zebrafish each before exclusions. Control and infrasound group zebrafish were tested in 4:1 alternating trials to ensure testing under identical conditions. EthoVision XT (v. 11, Noldus, VT, USA) motion tracking software was used to record behavioural responses and locomotion during the 5-min acclimation and experimental periods. Dependent variables analysed were distance moved, immobility, time in zone 1 (speaker), time in zone 2 (middle), time in zone 3 (far), and time in the thigmotaxis zone within acclimation and infrasound trials.

### Statistical analysis

Zone preference was tested using a repeated measures Three-way ANOVA to determine the effects of *exposure* (acclimation vs. infrasound), *zone* (speaker vs. middle vs. far vs. thigmotaxis), and *frequency* (0 Hz vs. 5 Hz vs. 10 Hz vs. 15 Hz vs. 20 Hz) on time spent in zones. Group means of distance moved, immobility, and time in each zone were calculated for both acclimation and experimental trials, along with respective standard deviations, standard errors, and 95% confidence intervals. Normality was determined using the D’Agostino-Pearson normality test. For locomotor variables (distance moved and immobility), parametric data was tested using a One-way ANOVA and Tukey’s multiple comparisons test or *via* a Kruskal–Wallis Test with Dunn’s Multiple comparison for non-parametric data. All data were analysed with GraphPad Prism (Version 9.0; GraphPad, San Diego, CA, USA) software. A significance level of 5% (α = 0.05) and a confidence interval of 95% were chosen to determine statistical significance of test values. Zebrafish that showed no movement for over 100 s were considered *immobile* and excluded from analysis. This exclusion criteria was based on previous studies in which immobile fish otherwise produced outliers and/or yielded no significant differences between groups (Holcombe et al., 2013; Szaszkiewicz et al., 2021) and on the distinction noted by Kalueff et al. (2013) between immobility and stress-induced freezing behaviours. It was determined that fish that are immobile are not necessarily experiencing stress, nor are they making a measurable choice whether to avoid or prefer a stimulus. Four fish from the 0 Hz group, 7 fish from the 5 Hz group, 6 fish from the 10 Hz group, 4 fish from the 15 Hz group, and 2 fish from the 20 Hz group were considered immobile and excluded from analysis. After exclusions, the study population size was *n* = 132 zebrafish. Data from EthoVision is available in the Supplementary materials section.

## Results

### Zone preference—speaker, middle, far, and thigmotaxis zones

A residual analysis of the group distributions showed that assumptions of normality, homogeneity of variances, multicollinearity, and homoscedasticity were met. No significant outliers were detected within the distributions. It was concluded that our repeated measures three-way ANOVA model was a good fit for the zone preference data.

After conducting the repeated measures three-way ANOVA, a significant main effect was found for *zone* location [*F*_(3,1056)_ = 948.141, η*^2^* = 0.7293, *p* < 0.001] on time in zones and no significant main effects were found for *exposure* [*F*_(1,1056)_ = 2.429, η*^2^* = 0.0023, *p* = 0.119] or *frequency* [*F*_(4,1056)_ = 0.480, η*^2^* = 0.0018, *p* = 0.750] on time in zones. Significant two-factor interaction effects were found for exposure*zone [*F*_(3,1056)_ = 6.446, η*^2^* = 0.0180, *p* < 0.001] and frequency*zone [*F*_(12,1056)_ = 3.718, η*^2^* = 0.041, *p* < 0.001] on time in zones but not for exposure*frequency [*F*_(4,1056)_ = 0.044, η*^2^* < 0.001, *p* = 0.996]. No significant three-factor interaction effect was observed for exposure*frequency*zone [*F*_(12,1056)_ = 0.965, η*^2^* = 0.011, *p* = 0.480] on time in zones. In summary, a large but expected significant effect was found for *zone* location on time in zones, a mild effect was found for *zone* location dependent on infrasound *exposure* on time in zones, and a moderate effect was found for *zone* location dependent on infrasound *frequency* on time in zones.

**FIGURE 2:**
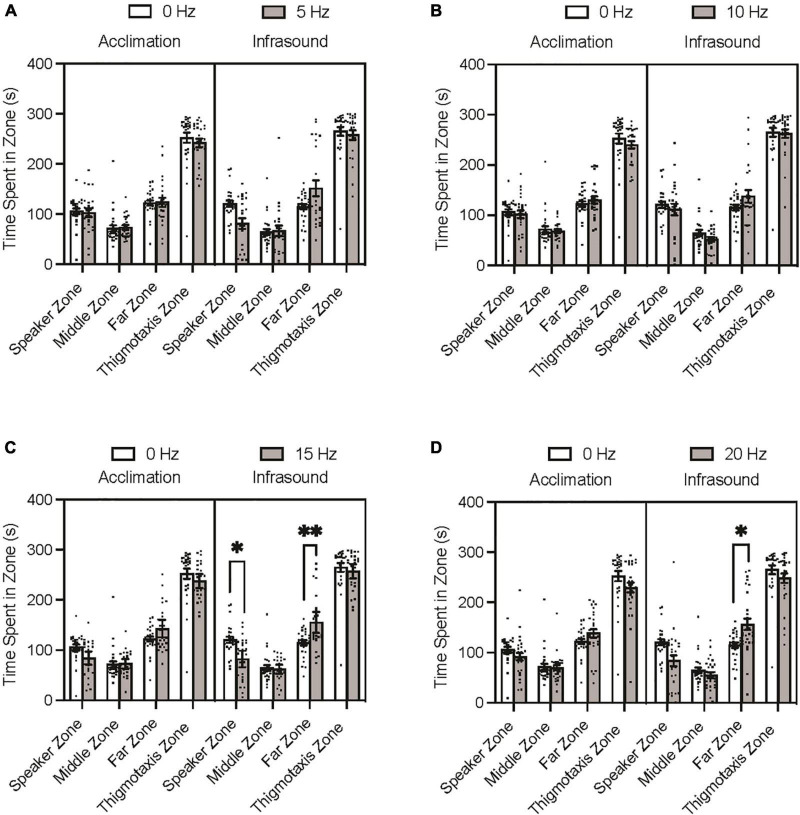
Zebrafish zone preference during acclimation and infrasound exposure trials. Fish were individually placed in the arena prior to the infrasound stimulus to record their baseline behaviour and then exposed to their respective frequency. **(A)** Average time spent in zones by 0 vs. 5 Hz fish. **(B)** Average time spent zones by 0 vs. 10 Hz fish. **(C)** Average time spent in zones by 0 vs. 15 Hz fish. **(D)** Average time spent in zones by 0 vs. 20 Hz fish. Data points represent individual zebrafish. Error bars represent S.E.M. *p < 0.05, **p < 0.01.

The Tukey’s multiple comparisons post hoc test indicated that none of the experimental groups differed significantly from the control group during the acclimation period trials on time spent in any of the zones (*p* > 0.05). During the infrasound exposure period trials, none of the experimental groups differed significantly from the control group on time spent in the *middle* zone (*p* > 0.05) or *thigmotaxis* zone (*p* > 0.05). The 5 Hz fish spent less time in the *speaker* zone and more time in the *far* zone than the control group during infrasound exposure, but after adjusting for familywise comparison these differences were not significant (Figure 2A; *p* = 0.0717; *p* = 0.1350). The 10 Hz fish did not differ significantly from the control group prior to or after adjustment for familywise comparison on time spent in the *speaker* and *far* zones during infrasound exposure (Figure 2B; *p* > 0.999; *p* = 0.7558). The 15 Hz fish spent less time in the *speaker* zone and more time in the *far* zone than the control group during infrasound exposure, and after adjusting for familywise comparison these differences were found to be statistically significant (Figure 2C; *p* = 0.0123; *p* = 0.0046). The 20 Hz fish spent less time in the *speaker* zone and more time in the *far* zone than the control group during infrasound exposure, and after adjusting for familywise comparison the difference in the *speaker* zone was not significant (*p* = 0.0708) but the difference in the *far* zone was significant (*p* = 0.0192) (Figure 2D). In summary, the 15 Hz and the 20 Hz groups spent significantly more time in the *far* zone than the control group during infrasound exposure. Only the 15 Hz group spent less time in the *speaker* zone than the control group during infrasound exposure.

### Locomotion—distance moved and immobility

**FIGURE 3:**
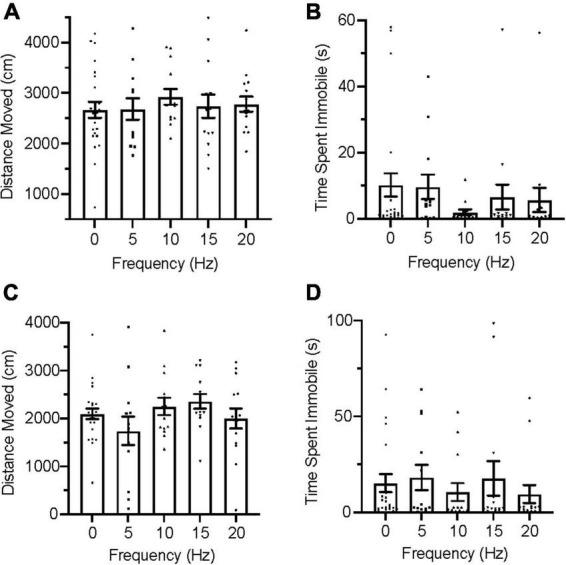
Zebrafish locomotion during acclimation and infrasound exposure. **(A)** Average distance moved during the acclimation period. **(B)** Average time spent immobile during the acclimation period. **(C)** Average distance moved during infrasound exposure. **(D)** Average time spent immobile during the infrasound exposure period. Data points represent individual zebrafish. Error bars represent S.E.M.

During the acclimation trials, all group distributions passed the D’Agostino and Pearson test for normality on measures of distance moved but failed it on immobility. An ordinary one-way ANOVA was used for distance moved and a Kruskal–Wallis test was used for immobility to evaluate group for differences. No significant differences were found between groups in distance moved [Figure 3A; *F*_(4,127)_ = 1.769, *p* = 0.1391], nor were any significant differences found between groups in immobility [Figure 3B; *H*(5) = 4.333, *p* = 0.3628] during acclimation.

During the infrasound exposure trials, group distributions for both distance moved and immobility failed the D’Agostino and Pearson test for normality and nonparametric Kruskal–Wallis tests were conducted for each measure of locomotion. No significant differences were found between groups for distance moved [Figure 3C; *H*(5) = 6.911, *p* = 0.1407] and there were also no significant differences found between groups for immobility [Figure 3D; *H*(5) = 7.548, *p* = 0.1096].

## Discussion

This study demonstrated that zebrafish moderately avoided 15 Hz infrasound frequencies in an open field test and suggests that infrasound may be aversive to zebrafish. Consistent with previous research on infrasound sensitivity (Sand and Karlsen, 1986; Karlsen, 1992a,b) as well as the results of such frequencies on aversion in aquatic organisms (Enger et al., 1993; Sand and Karlsen, 2000; Sand et al., 2001; Karlsen et al., 2004; Bui et al., 2013), infrasound tonal frequencies of 15 Hz significantly increased the amount of time spent in the far zone, furthest from the tone source, and decreased the amount of time spent in the speaker zone, closest to the tone source. The lack of a significant differences in time spent in the middle zone indicates that the fish moved away from the speaker zone in favour of the far zone and did not simply migrate away from the speaker with no preference between the two remaining zones. It can be inferred from this that fish in this condition experienced aversion, preferring to stay further away from the infrasound source. This is supported by the zone-dependent mild and moderate effects found for infrasound exposure and frequency, respectively, on zone preference. However, while aversion was observed, all groups spent similarly elevated amounts of time in the thigmotaxis zone and evidence of a distinct increase in anxiety-like behaviour could not be established at 15 Hz (or any other frequencies). The 20 Hz group spent significantly more time in the far zone but not significantly less time in the speaker zone and thus aversion cannot be inferred from this group difference. It should also be noted that all groups spent consistently less time in the middle zone than the speaker or far zones. Given that all fish spent a large proportion of time in the thigmotaxis zone, this is not surprising as the middle zone contains the least thigmotaxis area.

Previous studies on infrasound and fish have used experimental apparatuses based on Sand and Karlsen (1986) with pistons and vibrators causing displacement of the entire water column. Our approach used generation of infrasound in air, which approximates some forms of infrasound pollution in urban settings. This is more relevant to aquatic housing areas within laboratories that are surrounded by human infrastructure. In our experiments zebrafish spent less time near the infrasound source which indicates that under our experimental conditions the presence of 15 Hz from an air-originated source elicits an aversive response. Future research may be needed to assess the environmental noise pollution of human industry and technologies on aquatic organisms in both natural and artificial habitats. Identification and subsequent mitigation of any high amplitude 15 Hz frequencies in domestic and laboratory environments containing aquatic species may also contribute to their habitat enrichment and wellbeing.

Aversion responses observed in zebrafish in this study are consistent with observations of Atlantic Cod (Sand and Karlsen, 1986, 2000), Perch (Karlsen, 1992b; Sand and Karlsen, 2000), Plaice (Karlsen, 1992a), and Atlantic Salmon (Sand et al., 2001). Each of these species, like zebrafish, were found to exhibit avoidant responses to frequencies between 0.1 and 20 Hz. Given this moderately generalized response, it is possible that this avoidant behaviour to infrasound frequencies is an evolutionarily conserved mechanism that may be found in all teleost species of fish. Our results differed slightly from some previous literature in the exact frequency—or range of frequencies—that produced the strongest avoidance responses. Zebrafish avoided 15 Hz frequencies in our study, while Atlantic Cod responded strongest to 12.5 Hz (Bui et al., 2013) and Atlantic Salmon to 5–10 Hz (Sand et al., 2001). It is possible that species-specific variances in infrasound frequency responsiveness may exist in relation to organism size and, by extension, the size of their natural predators. Zebrafish also did not show behaviours indicative of a startle response, as was found in *Rutilus rutilus* (Karlsen et al., 2004), or fear-like behaviour, as seen in Plaice (Karlsen, 1992a) that would indicate anxiety.

These findings lead to many unanswered questions: (1) Given that 5 Hz increments of infrasound stimuli were used in this study, future studies should further fine tune the spectrum of response with smaller increments, with a focus on 12.5 Hz. This frequency may elicit anxiety responses similar to other teleost fish like Atlantic cod (Bui et al., 2013). (2) Infrasound exposure may yield more pronounced and generalized effects, including anxiogenic properties, in exposure periods of longer than 5 min, as would be the case in laboratory habitats. Future studies could thus explore the dynamics of these effects as a function of infrasound exposure duration. (3) Our infrasound validation measurements were taken from the air above the arena and not within the water itself. Though the frequency (or frequencies) of a sound is generally considered constant between gaseous and fluid media due to the continuity of soundwave motion, future studies could expand on this design by exploring the physical dynamics of differing infrasound source locations and media. (4) The experimental trials in this study used a constant amplitude of ∼60 dB; future research could explore frequencies at amplitudes above or below this magnitude. Together, these directions of future study (1–4) would contribute to our understanding of how infrasound may act across different amplitudes, media, and sources. Furthermore, future studies could use more complex paradigms of aversion—such as in conditioned behaviour studies—to evaluate both the validity of this phenomenon as well as its generalizability to other environments and aversion-related behaviours.

The effects of infrasound appear to be shared across many teleost species. Given the findings of this study, it is possible that zebrafish could be used as a model organism in future research on the effects of infrasound on other similar species, both freshwater and marine. Infrasound frequencies have been recorded from marine wind turbines, peaking at approximately 15 Hz during wind velocities between 5.2 and 5.67 m/s (Boczar et al., 2022), which parallel peak frequencies of aversion in our study. Some diesel engines have also been found to generate infrasound (Persinger, 2013), of which a wide variety are used in marine industry. Sand et al. (2001) demonstrated that infrasound frequencies may be used as a deterrent method for keeping fish away from areas containing marine industrial infrastructures and operation. It follows that infrasound frequencies could also have potential to cause unintentional disruption of marine habitats. Future studies could explore zebrafish as a model organism of infrasound-induced aversion in broader environmental studies evaluating the effects of sound pollution on both wild freshwater and marine fish.

Lastly, these findings indicate that infrasound frequencies of 15 Hz could be used as a valid stressor in behavioural testing of aversion in zebrafish. Previous studies have used a variety of stressors including olfactory alarm cues, electric shocks, temperature and light level changes, ultraviolet light, mechanical environmental disturbances, and anxiogenic drugs [e.g., Bai et al., 2016 in larval zebrafish; Egan et al. (2009) and Giovana et al. (2022) in adult zebrafish]. The advantage of infrasound as an aversive stimulus is that it can be administered in a non-invasive and painless manner and quickly turned on or off, in contrast to immersion in an olfactory alarm cue or anxiogenic substance that take time to be eliminated. Additionally, anxiety models using electric shocks in other teleost fish, such as Atlantic Cod, Plaice, and Perch in much of Sand and Karlsen’s (1986; 2000) and Karlsen’s, (1992a,b) infrasound research, require not only anaesthesia and physical placement of electrodes on fish for physiological measurement, thus complicating the experimental design. Using infrasound as a stressor does still require further validation using anxiolytic compounds to examine whether ‘stress’ by infrasound can be predictably mitigated. However, there is potential for use as a valuable tool in the fields of behavioural neuroscience and psychoacoustics.

## Data availability statement

The original contributions presented in this study are included in the article/Supplementary material, further inquiries can be directed to the corresponding author.

## Ethics statement

The animal study was reviewed and approved by MacEwan University’s Animal Research Ethics Board (AREB) under protocol 05-12-13, in compliance with the Canadian Council on Animal Care (CCAC) guidelines for the care and use of experimental animals.

## Author contributions

TH, TE, and RS designed the experiment. TP and KS conducted the experiment and collected the data. KS performed the statistical analysis and interpretation of the data and wrote the main manuscript. TH, RS, and KS edited the manuscript and prepared the study figures. All authors contributed to the article and approved the submitted version.

## Footnotes

1
www.cdc.gov
